# A research roadmap for SCN8A-related disorders: addressing knowledge gaps and aligning research priorities across stakeholders

**DOI:** 10.1186/s13023-025-03672-w

**Published:** 2025-08-19

**Authors:** The SCN8A Research Consortium, Anne T. Berg, Christopher Burge, Gabi Conecker, Dylann Cordova-Martinez, Cristine Cukiert, Andrew Escayg, Mark Fitzgerald, Elena Gardella, Joshua Hack, Michael F. Hammer, Chellamani Harini, JayEtta Hecker, Dennis Lal, Christopher D. Makinson, Ian Miller, Kelly Muzyczka, Rima Nabbout, Madeleine Oudin, Manoj Patel, Steve Petrou, Jeremy Prokop, Alex Rotenberg, Ingrid Scheffer, John M. Schreiber, Jennifer Wong, Wenxi Yu

**Affiliations:** Tucson, AZ USA

**Keywords:** Pediatric epilepsy, Voltage-gated sodium channnel, Disease spectrum, Knowledge gaps, Research priorities, Stakeholders, Caregivers, Disease management, Disease mechanism

## Abstract

**Background:**

Despite significant scientific progress since the 2012 discovery that variants in the *SCN8A* gene can cause human epilepsy, disease mechanisms and best practices for management of SCN8A-related disorders (SCN8A-RD) remain incompletely understood. To accelerate the rate of progress, the *International SCN8A Alliance* sponsored a conference in Boston, Massachusetts, on August 16–18, 2024. The goals were to identify core knowledge gaps and research priorities, and to establish a collaborative research strategy to improve quality of life. In addition to a number of family leaders representing caregiver priorities, the meeting included laboratory scientists, clinicians, and representatives from the biopharmaceutical industry.

**Main body:**

The scientific literature and requests for proposals from epilepsy funding agencies were reviewed prior to the meeting. Stakeholder-specific surveys were conducted focusing on knowledge gaps, research priorities, and scientific roadblocks. Interviews with biotechnology leaders were conducted to identify their priorities. These data were analyzed to assess responsiveness to caregiver concerns and to identify top research priorities for advancing the field. The Caregiver survey (n = 175) revealed top challenges and identified novel therapeutics and management of non-seizure phenotypes/comorbidities as top priorities. Clinician (n = 46) and scientist (n = 23) surveys identified a number of common research priorities, partially overlapping with caregiver concerns. Five core areas emerged from integrated analysis of all four stakeholder surveys and became the focus areas of five *Working Groups*: (1) Transformative Therapeutics, (2) Non-Seizure Outcomes, (3) Current Therapeutics, (4) Biomarkers, and (5) Whole Brain/Whole Body.

**Conclusions:**

Taking account of the concerns and priorities of the caregiver community, the five working groups identified research directions to address knowledge gaps that include both short- and long-term priorities to improve understanding of disease mechanisms and management for the spectrum of SCN8A-RD phenotypes. Challenges included identification of suitable funding mechanisms and the lack of expertise in certain methodologies and research areas. This Research Roadmap is expected to accelerate progress toward the goals of improved quality of life and transformative care for all those with SCN8A-RD.

**Supplementary Information:**

The online version contains supplementary material available at 10.1186/s13023-025-03672-w.

## Introduction

Pathogenic variants in the voltage-gated sodium channel gene, *SCN8A* (encoding Na_V_1.6), underlie a wide spectrum of clinical phenotypes ranging from neurodevelopmental delays (NDD) with or without seizures—to severe developmental and epileptic encephalopathy (DEE). While Na_V_1.6 was known to play a critical role in neuronal excitability since the mid-1990’s [1, 2], it wasn’t until 2012 that the first pathogenic *SCN8A* variant was identified in a child with DEE and sudden unexpected death in epilepsy (SUDEP) [3]. Soon thereafter the *SCN8A* gene was included on commercial epilepsy gene panels and an online patient registry (the *International SCN8A Registry*) was established [4, 5].

## Brief history of progress

### Case reports, cohort and registry studies

The majority of initial cases experienced severe DEE, characterized by early onset seizures, global developmental delay, intellectual disability, and motor function impairment [6–8]. As an increasing number of case reports and cohort studies were published the disease spectrum widened to include patients with milder forms of epilepsy and neurodevelopmental delays (NDD) without seizures [7, 9–17], including patients with gain of function (GoF), loss of function (LoF) and mixed GoF/LoF variants [15, 18–21]. Studies making use of large cohorts and *Registry* data identified genotype–phenotype correlations [9, 14, 15, 19, 22, 23], as well as phenotypic subgroups [10, 15, 24]. The application of machine learning (ML) methods to classify *Registry* participants into phenotypic subgroups based on early clinical features resulted in promising new methods to inform treatment decisions [18] and improve prognosis [24, 25].

### Incidence and prevalence

In a recent survey of autosomal dominant de novo monogenic rare neurodevelopmental disorders, SCN8A-RD had an estimated prevalence of 2.96/100,000 individuals (95% CI 2.63–3.24/100,000 individuals) [26]. The incidence of SCN8A-RD is reported to be just over 1 in 56,000 births [15]. Based on the 2021 Centers for Disease Control and Prevention data [27], we infer a birth rate of ~ 65 SCN8A-RD cases per year in the United States (U.S.). There are known to be over 700 cases worldwide with pathogenic variants in *SCN8A*, accounting for ~ 1% of all cases of epilepsy with encephalopathy [15].

### Consensus guidelines for diagnosis, phenotypes, and treatment

Beginning in 2021, the *International SCN8A Alliance* (*Alliance*) convened a core panel of more than 30 experts to develop the first global consensus for diagnosis and management of SCN8A-RD. The panel investigated the extent of agreement in a modified-Delphi process that addressed diagnosis, treatment, phenotypes, comorbidities, and clinical management [28], as well as comorbidities and prognosis [29].

### Preclinical models: mechanisms

Preclinical and laboratory models for SCN8A have been instrumental in advancing understanding of disease mechanisms and facilitating therapeutic development for SCN8A-RD [30, 31]. In vitro models, including heterologous expression systems and patient-derived induced pluripotent stem cells (iPSCs) differentiated into neurons, have enabled detailed electrophysiological studies of mutant Nav1.6 channels [20, 32–34]. Genetically modified mice and zebrafish models have also been used [35, 36]. Mouse models, such as constitutive and conditional knock-in mice carrying human *SCN8A* mutations, have been crucial in identifying disease mechanisms, including sex differences [31, 37–42]. These diverse models have yielded significant insights into the altered channel kinetics and neuronal hyperexcitability associated with mutant SCN8A and elucidated the role of Na_V_1.6 in different neuron types and brain regions [43, 44].

### Therapeutics

Several studies have been instrumental in identifying treatments for GoF-associated SCN8A-RD: with the development of novel small molecule inhibitors that directly target Na_V_1.6, use of modulators of neuronal excitability, and the repurposing of existing medications [34, 45–48]. Preclinical studies have demonstrated that reduction in the levels of *Scn8a* transcripts with GoF variants by antisense oligonucleotides (ASOs) can prevent seizures and extend survival in a *Scn8a*-DEE mouse model [49], even when treatment is initiated after seizure onset [50]. Down-regulation of *Scn8a* by viral delivery of an *Scn8a*-shRNA was also seizure protective [51]. In addition, allele-specific inactivation of the mutant allele by CRISPR/Cas9 also rescued dominant lethality in *Scn8a*^*N1768D*^/ + mice [52]. While promising as a therapeutic strategy for variants with GoF effects, there are currently no published preclinical studies on the potential of small molecule or genetic therapies in the context of *SCN8A* LoF variants.

### Industry advances in clinical trials

There are currently two small molecules in clinical trials for GoF variants in *SCN8A* that lead to excess Na_V_1.6 sodium current: NBI-921352, which directly targets Na_V_1.6 channels [45] (NCT04873869) and PRAX-562 [53], which primarily targets persistent sodium currents in both Na_v_1.6 and Na_v_1.2 (NCT05818553).

## Knowledge gaps and critical barriers to progress

Despite these gains, critical knowledge gaps remain in the basic understanding of disease mechanisms and clinical management. The relationships between in vitro and in vivo functional effects for *SCN8A* variants [54] are poorly understood and there are currently no known biomarkers to track disease progression or treatment response. Treatment approaches to the multifarious manifestations of the disease are hindered by the (1) non-selective mechanisms of currently used sodium channel blockers (SCB), (2) paucity of knowledge on polypharmacy, (3) poor understanding of the relationships between epilepsy and non-seizure outcomes [7, 55], and (4) lack of therapeutic choices for managing non-seizure comorbidities and the high premature death rate (Table 1). Furthermore, efficient clinical trial design and the production of orphan products is stymied by: (1) incomplete knowledge of disease natural history; (2) selection of appropriate outcome measures for the full disease spectrum; and (3) a “floor” effect on existing developmental scales that limits application to the most severely impacted patients [7].

**Table 1:** Critical knowledge gaps

Disease mechanisms	Mechanisms of epileptogenesis
	Relationships between in vitro and in vivo functional effects for SCN8A variants
	Relationships between epilepsy and non-seizure outcomes
	Lack of biomarkers to track disease progression or treatment response
Clinical management	Non-selective mechanisms of sodium channel blockers
	Use of polypharmacy
	Therapies for non-seizure comorbidities
	Prevention of premature death
Clinical trial design	Disease natural history
	Outcome measures for the full disease spectrum
	Floor effect of developmental scales for severely affected

## The SCN8A research roadmap meeting

The *Alliance* sponsored an *SCN8A* Research Roadmap meeting that was held in Boston, MA on August 16–18, 2024, with the overarching goal to rapidly advance the field, taking into consideration caregiver priorities for improved quality of life (QoL). The meeting brought together caregivers, clinicians, scientists, and biotechnology industry partners to (1) identify knowledge gaps in the mechanisms of epileptogenesis and a multitude of non-seizure outcomes, (2) prioritize research strategies to fill these gaps, and (3) promote collaborative efforts among stakeholders to simulate the development of therapies with potential to have the largest impact for improved QoL.

## Stakeholder survey results

### Survey design

Extensive review of the SCN8A literature (more than 460 publications since 2012) and requests for proposals from epilepsy funding agencies were performed (e.g., CURE, Epilepsy Foundation, NINDS). Stakeholder-specific surveys were crafted focusing on research priorities, scientific roadblocks, current gaps in research and clinical care. Interviews with biotech leaders were also conducted to identify priorities from the biopharma perspective. One of the meeting organizers (CBB) interviewed 8 biotech companies interested in SCN8A. Full survey results can be found in Supplemental Material and are briefly summarized below.

### Caregivers

**Fig. 1:**
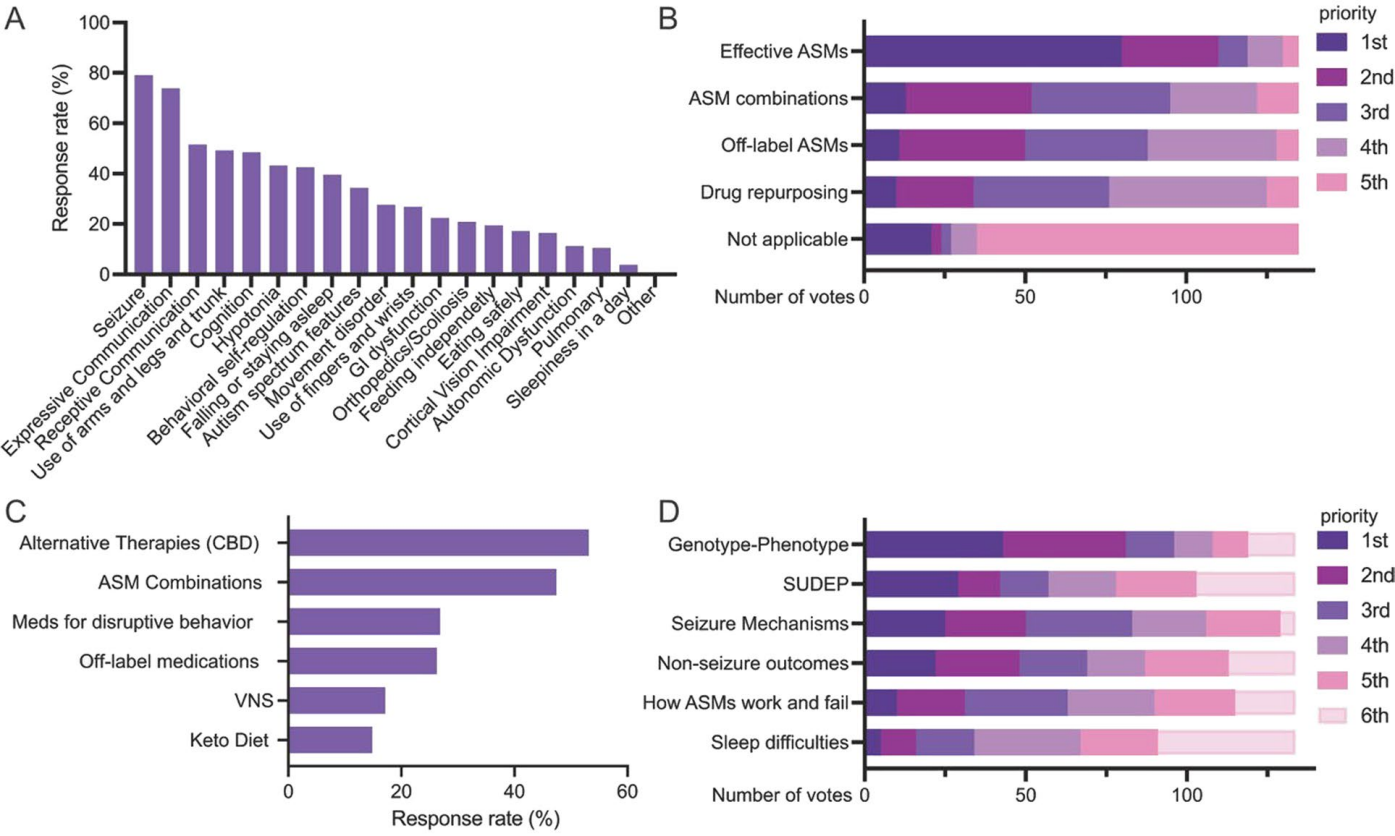
Caregiver survey responses. **A** Rankings of the top five challenges affecting quality of life (QoL), **B** priorities for controlling seizures, **C** interventions warranting further investigation, **D** priorities for research into disease mechanisms

The caregiver survey received responses from 175 families living in 27 countries. The top five challenges that affected QoL involved seizures, cognition and speech, motor disabilities (including hypotonia), sleep and behavioral issues (Fig. 1A). Behavioral issues were more commonly a top challenge for caregivers of patients with LoF variants. Caregivers prioritized research to improve seizure control over that for non-seizure outcomes by 60:40. To control seizures, caregivers prioritized research on the development of new effective antiseizure medications (ASMs) (Fig. 1B), followed by alternative therapies (e.g., CBD), and ASM combinations (Fig. 1C). Regarding research into disease mechanisms, caregivers gave the top rank to research that would increase understanding of the effect of individual genetic variants on disease phenotype or severity (Fig. 1D).

### Clinicians

**Fig. 2:**
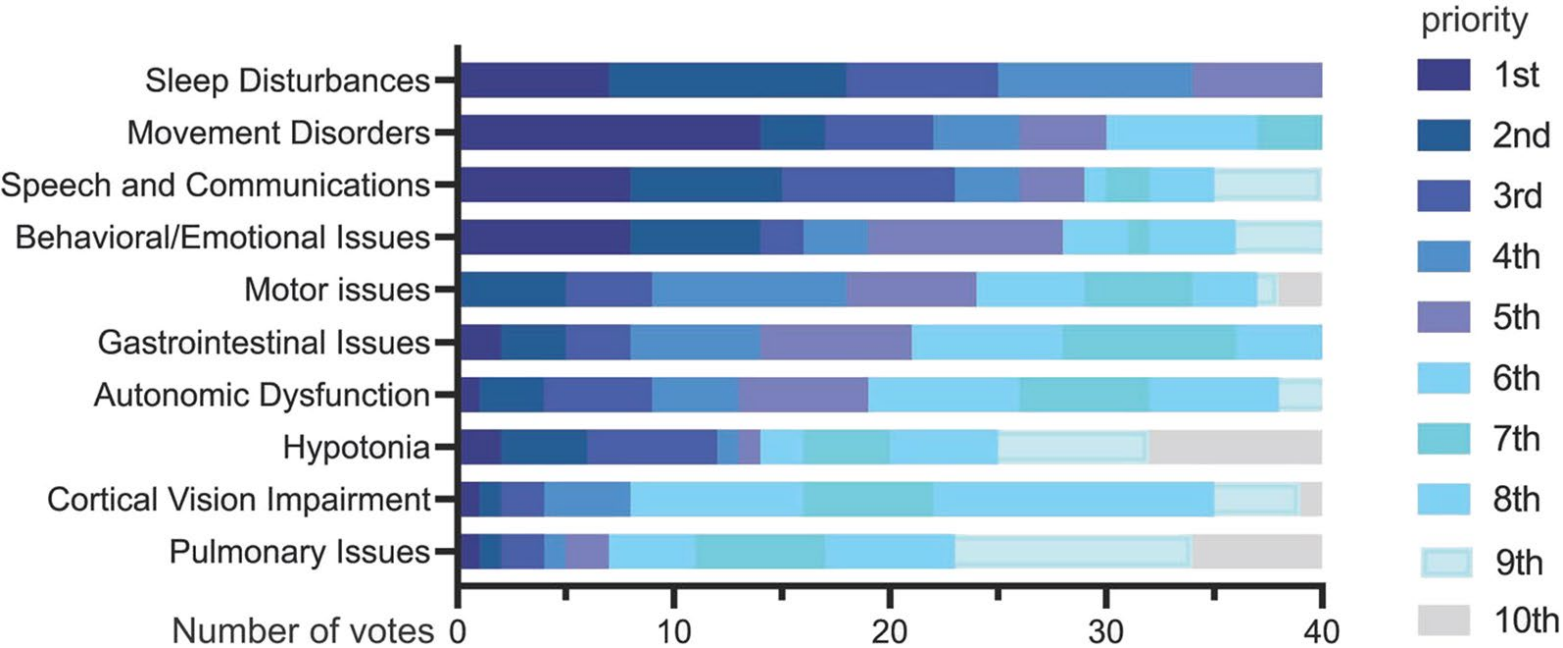
Clinician ranking of importance of research on 10 comorbidities to improve QoL

Clinicians (n = 46) were asked about clinical experience and expertise in SCN8A-RD (Figs S1A and S1B). They emphasized the importance of detailed characterization of the phenotypic spectrum to improve diagnosis and phenotyping (Fig S2A). To improve treatment, clinicians prioritized research to guide selection of first line and adjunctive ASMs (their doses and combinations) (Fig S2B). To improve prognosis, clinicians prioritized the identification of features that predict clinical outcome (Fig S2C). Regarding research to improve QoL, clinicians prioritized movement disorders (e.g., ataxia) (30.4%), followed by speech/communication and behavioral issues (17.4%) (Fig. 2). Clinicians also prioritized registry-based longitudinal research and prospective natural history studies to advance the ability to accurately diagnose, predict outcomes, and tailor treatments (Fig S2D–F).

### Scientists

Scientists (n = 23) with expertise in a variety of subfields participated in the survey. This group had a median of 5.5 years in SCN8A-related research with a median of 20% effort. They reported nearly equal feasibility in studying GoF and LoF mechanisms (Fig S3A) and greater feasibility for studies of seizure-related *versus* non-seizure-related mechanisms in their respective labs (Fig S3B).

**FIg 3:**
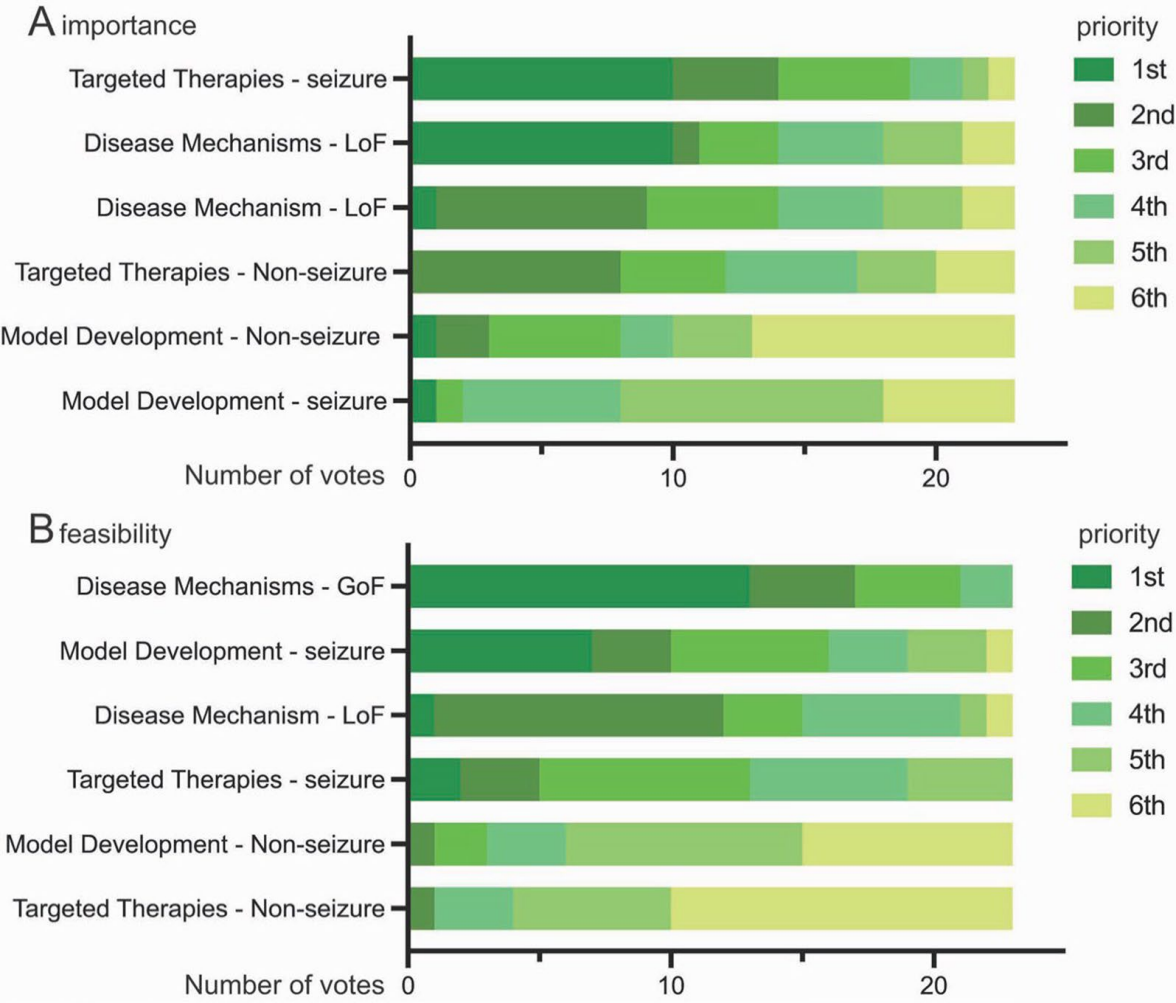
Scientist ranking of research goals to fill knowledge gaps: Importance (**A**) versus feasibility (**B**) of current models

Overall, the scientists ranked the development of targeted therapies for seizures as the most important, followed by investigating the mechanisms driving GoF and LoF phenotypes (Fig. 3A). Importantly, there was a different rank order for the feasibility of accomplishing these goals (Fig. 3B). For instance, developing targeted therapies for seizures was ranked 1st in importance but 4th in terms of feasibility. Model development to study seizures was ranked lowest in terms of importance but highest in terms of feasibility given the current availability of seizure models for SCN8A-RD.

Scientists ranked molecular and cellular-based research on seizures as the top priority to understand disease mechanisms (Fig S3C). Regarding improved understanding of mechanisms to improve therapeutics, research into whole brain circuitry and the development of biomarkers were ranked as top priorities (Fig S3D). ASOs were ranked as the top research priority to develop and deliver genetic therapies for variants with both GoF and LoF properties (Fig S4A). The "Goldilocks" problem (i.e., disease phenotypes caused by ‘too little or too much’ Na_V_1.6 activity) was ranked the most critical issue to overcome in order to successfully develop targeted genetic therapies (Fig S4B).

To improve clinical outcomes, scientists reached a strong consensus on the importance developing laboratory models for the purpose of designing and delivering selective Na_V_1.6 therapies (e.g., novel small molecules), specifically transgenic mouse models (Fig S5A, B). For the purpose of filling knowledge gaps in disease mechanisms in relation to therapeutics (i.e., testing selective ASMs and genetic therapies), transgenic mouse models also ranked as the most important model type (Fig S5C). The survey also emphasized the importance of developing models to study non-seizure phenotypes (Fig S5D); however, scientists also acknowledged limitations of current animal models in recapitulating comorbidities commonly observed in patients and the challenge to develop such models (Fig. 3).

### Biotech perspective

Companies are drawn to drug development for SCN8A-RD for a variety of reasons, including a clear unmet medical need, the well-organized patient community, and the collective expertise of scientists and clinicians in CNS and ion channels. Challenges for biotech include the rarity of the disease and the crowded space of existing drugs, which complicates clinical trial design and execution. Similar to GoF strategies favored by scientists, biotech favored approaches that would inhibit channel activity, knockdown expression, and promote inhibitory neuron activity. Favored approaches in the case of LoF included blocking "poison exons" or microRNA target sites with ASOs, and potentially using CRISPR activation. Biotech also acknowledged challenges in the risk of excessive knockdown, the need for delivery to relevant brain regions, and the need to classify patients as carrying variants with GoF or LoF function based on clinical phenotype.

Priorities for research that would help de-risk investment included better classification and understanding of variant biology and pathophysiology, determination of the range of Na_V_1.6 levels compatible with brain health, development of additional animal models for various variant classes, and implementation of natural history studies that do not preclude clinical trial participation. Companies also expressed interest in more information on phenotype-genotype correlations and the development of an iPSC resource managed by an organization like ATCC. Collaboration opportunities were also identified, including increased interaction with other channelopathies (e.g., SCN2A) and assistance in determining measurable clinical trial endpoints.

## Integration of survey results

### Compare and contrast caregiver, clinician and scientist surveys

#### Points of agreement

All stakeholder groups prioritized improving seizure control as a primary research goal. There was also a shared interest in advancing genetic therapy approaches, which are seen as promising avenues for targeted treatment. All groups also recognized the importance of studying both seizure and non-seizure outcomes, as well as the need for more personalized treatment strategies (e.g., tailored to specific variants and/or their effects on Na_V_1.6 activity). Stakeholders also were consistent in the recognition of the need for more data collection and interdisciplinary collaboration (Table 2).

**Table 2:** Points of agreement among stakeholders and responsiveness to caregiver priorities

Priority area (alignment)	Points of agreements	Clinician responsiveness	Scientist responsiveness
Seizure vs. Non Seizure Outcomes (Partial)	All recognize importance of both; caregivers prioritize non-seizure outcomes, clinicians and scientists focus more on seizure control	Recognize importance of comorbidities, seizures remain top priority	Primary focus on seizure therapies
Comorbidities (High)	All recognize importance	Prioritize sleep, movement, and behavioral issues	Recognize importance, not primary focus
Genetic Therapies vs. Conventional ASMs (Partial)	Caregivers strongly prefer research into genetic therapies, clinicians prioritize ASMs, scientists focus on both	Prioritize traditional ASM approaches over genetic therapies	More focus on genetic therapies then on other targeted treatments
Novel ASMs (High)	All see importance, different emphasis	Top priority, first line and adjunctive top priority	Focus on novel small molecules repurposed drugs
Research Models (Partial)	Agreement on natural history studies, different emphases; Caregivers interested in participating in studies, scientists focus on lab models	Align well with caregiver interest in participation; Emphasize registries and digital health technology involving patients	Focus on transgenic mouse models (do not directly involve patients), less on patient-involved studies

#### Caregiver priorities

In contrast to scientists and clinicians, caregivers tended to prioritize immediate, practical interventions to improve daily life, including alternative therapies (e.g., Fig. 1C vs Figs S2B and S5A). While all groups recognized the importance of non-seizure outcomes and comorbidities, caregivers placed higher importance on cognitive impairment, communication difficulties, and behavioral problems; clinicians prioritized sleep, movement, and behavioral issues; and scientists focused more on cognitive aspects and SUDEP. Clinicians also recognized the importance of research on non-seizure phenotypes, while scientists prioritized research on fundamental mechanisms, particularly through the use of cellular and animal models.

#### Research priorities of clinicians and scientists

Disease mechanisms and treatment development represent top research priorities for both clinicians and scientists (Table 3). Both groups also recognized the importance of biomarkers as crucial research tools for understanding disease mechanisms and the impact of therapeutics. The value of registries and natural history studies represents another area of strong alignment, as was the importance of interdisciplinary collaboration and partnerships between clinics and laboratories. While both groups prioritized seizure control, clinicians tended to emphasize research on first line and adjunctive ASMs, while scientists focused on selective Na_V_1.6 therapies, repurposed drugs, and genetic therapies. In terms of research tools, scientists placed emphasis on transgenic mouse models for testing therapeutics, while clinicians focused on the development of clinical data pipelines and predictive modeling.

**Table 3:** Points of Agreement on Research Priorities of Clinicians and Scientists

Research area	Clinician priority	Scientist priority	Agreement level
Disease Mechanisms	Recognized as Important research area	Top research priority	High
Treatment and Therapeutics	Top research priority (mostly ASMs)	Top priority (targeted therapies for seizures)	High
Genetic Therapies	Recognized as Important research area	Top research priority	High
Biomarkers	High priority for research tools and methods	Important research question for disease mechanisms and therapeutics	High
Registries and Natural History Studies	SCN8A registry as top priority for research tools	Recognized value of registry data to guide lab research	High
Electrophysiology and Neurophysiology	Recognized as crucial	Listed as key areas of expertise	High
Interdisciplinary Collaboration	Emphasized interclinic partnerships and multidisciplinary collaboration	Highlighted importance of lab clinic collaborations	High
Cell Types and Brain Regions	Implicit support through emphasis on disease mechanisms	Ranked as important for understanding epileptogenesis	Moderate
Pharmacology and Drug Development	Prioritized research on first line and adjunctive ASMs	Focused on selective Nav1.6 therapies and repurposed drugs	Moderate
Prioirtized Research (non-seizure)	Sleep, Movement, Speech, Behavior	Cognitive, behavioral, movement, SUDEP, hypotonia	Moderate
Non-Seizure Outcomes	Comorbidities recognized at important research area (patients)	Non-seizure phenotypes recognized as Important research area (models)	Partial
Research Tools	Electrophysiology, neurophysiology, predictive models, biomarkers	Transgenic mouse for testing therapeutics, Registry, Lab-Clinic collaboration, biomarkers	n/a
Prioirtized Research (seizure)	1st line ASMs & adjunctive, polypharmacy, off-label ASMs, repurposed drugs	Disease mechanism, ASO genetic therapy/ Goldilocks problem	Low

### Emergence of core knowledge gaps

Integration across stakeholder surveys yielded several core knowledge gap areas. Leaders with expertise in five core areas were identified and asked to represent the importance of each area to participants at the meeting. The following research areas were adopted to be the focus of five *Working Groups (WG)*:

WG1: Advancing Transformative Therapeutics: Development and Delivery
WG2: Non-Seizure Outcomes: Mechanisms, Management, and Therapies
WG3: Improving Current Therapeutics: First line, adjunctive, combination, and alternative approaches
WG4: Application of Biomarkers: Tracking disease progression and treatment response
WG5: Whole Brain/Whole Body: Brain regions, cell types, circuitry, neuromodulation & epileptic body

The working groups were assigned the following tasks: (1) define core knowledge gaps in each area, (2) design a research strategy to fill these knowledge gaps, and (3) ensure that the strategy facilitated improvement in patient outcomes and addressed caregiver priorities. Cross-disease comparisons (e.g., *SCN2A*) was suggested as a sixth area. Registries and natural history studies were also recognized as a core area of importance [56]. However, given the existence of a mature registry [4, 15] and ongoing efforts to initiate prospective natural history studies [57], this was not a focus of the Working Group sessions.

## Working group summaries

### WG1: advancing transformative therapeutics: development and delivery

#### Background

Current ASMs have limited effectiveness for the treatment of SCN8A-RD, with a significant portion of patients exhibiting refractory seizures [9]. Further, current ASMs do not address the wide range of cognitive and motor comorbidities that patients experience [29]. Transformative therapeutics that can manage refractory seizures and also improve non-seizure outcomes are needed. Both small molecules that directly target Na_V_1.6 or genetic therapies that directly correct the DNA or mRNA to restore protein function or alter he DNA or mRNA to mitigate deleterious effects caused by mutant protein could be transformative therapies. However, studies suggest that *SCN8A* is a “Goldilocks” gene, where too much or too little activity can cause abnormal phenotypes. In the case of GoF variants, only a small proportion of mutant transcript is sufficient to induce spontaneous seizures [58], while reduced expression or haploinsufficiency is associated with a range of symptoms such as seizures, ataxia, and intellectual disability [15, 18, 21, 29, 59].

#### Knowledge gaps

Given the Goldilocks nature of SCN8A, a major knowledge gap centers around levels of expression that are compatible with healthy brain function. Further research is required to identify safe and effective levels of *SCN8A* transcript that would restore normal neuronal activity without causing adverse effects. The lack of precise information also discourages biotech from investing in *SCN8A* drug development. Other areas needing further study concern the developmental stage(s) for optimal intervention, as well as the best physical location(s) in the brain to target. For example, *SCN8A* expression is low during early fetal development, increases approximately two months before birth, and plateaus at approximately one year of age [60]. Similarly, the effects of altering *SCN8A* expression in different circuits and brain regions has not been studied. Further, delivery methods will have to be optimized. Finally, it is uncertain whether therapies that directly target *SCN8A* in the CNS would lead to improved non-seizure outcomes.

#### Research priorities

This working group agreed that the top priority is to address the safety concern regarding excessive downregulation or upregulation of *SCN8A* expression. Varied levels of Scn8a expression can be introduced in wildtype mice by varying the doses of genetic therapeutics, such as ASO, CRISPR-KO, shRNA, and gene replacement followed by evaluation of seizure activity, motor function, behavior, cognitive function and survival. These studies should be carried out in whole brain and in specific brain regions of wildtype mice.

A second goal is to determine the effectiveness of genetic therapies in disease models of different severity with both LoF and GoF variants, and to determine the impact of timing of intervention during development. If early treatment has significant therapeutic advantages, these data will be useful for clinicians to advocate for early genetic screening, diagnosis, and intervention.

A third priority is to investigate the regulation of *SCN8A* expression, including transcriptional and epigenetic regulation. These studies could identify new targets and strategies for manipulating its expression, that could allow for more precise and tunable control over *SCN8A* expression in the CNS.

A fourth priority centers around the importance of non-seizure outcomes as highlighted in the caregiver survey. More research on existing mouse models is needed to assess the effects of new therapies on motor, behavior, cognition and other phenotypes. In addition, there is a need for new animal models that recapitulate the non-seizure phenotypes of importance in patients and that could be used for outcome measures in preclinical trials.

### WG2: non-seizure outcomes: mechanisms, management, and therapies

#### Background

Non-seizure morbidities are a significant concern in SCN8A-RD, with multiple studies showing high prevalence of symptoms such as communication and developmental delays, hypotonia, ataxia, and autistic-like behavior, as well as feeding, gastrointestinal and sleep difficulties (Fig. 1A). These are challenges that are often more concerning to caregivers than seizures themselves because of their enormous impact on QoL. Giving more attention to non-seizure challenges aligns with FDA guidance emphasizing the need to focus on disease features most important to patients in clinical trials.

#### Knowledge gaps

While non-seizure outcomes are becoming more recognized as significant challenges for SCN8A-RD families, there is still limited understanding of their causation and the best practices to manage them. Appropriate outcome measures are also needed to accelerate trial readiness. For example, a major gap is that current Clinical Outcome Assessments (COAs) are psychometrically unfit for use in SCN8A and other severely affected DEE populations, with existing measures like the VABS-3 Communication Scores showing extreme floor effects and decreasing standardized scores with age.

#### Research priorities

The working group agreed that a first priority is to comprehensively document the non-seizure outcomes adversely affecting the entire spectrum of patients with SCN8A-RD. This will involve collaborations with clinicians and families to create a detailed catalog of comorbidities, their prevalence, and their impact on QoL.

A second priority is to select appropriate outcome measures for non-seizure phenotypes. This requires a multidisciplinary approach, involving specialists from various fields such as neurology, developmental pediatrics, orthopedics, psychology, speech and language pathology, and occupational and physical therapy. It is important that these assessment tools capture the full spectrum of abilities and challenges in this patient population, from basic neurological function to adaptive behaviors. Consistent with the goals of the Inchstone Project [61], special attention should be given to finer-scale developmental measures that are more appropriated for severely affected individuals who may not show progress on traditional scales.

Given that many comorbidities are not unique to SCN8A-RD patients, a third priority is to collect data across channelopathies and DEEs to increase our understanding of comorbidities along the spectrum of neurodevelopmental disorders. This work will require strong collaboration between clinicians and caregivers. Leveraging existing data from multiple sources, including the PERC network [62], Ciitizen database [63], and the International SCN8A Patient Registry [4] will be essential to provide real-world evidence to inform which non-seizure phenotypes to prioritize based on both frequency and severity.

A fourth priority is to work with researchers who are involved in obtaining multisource data (see WG3) in standardized format to include non-seizure phenotypes with the goal of documenting the progression of these symptoms over time and how they are associated with seizures.

### WG3: improving current therapeutics

#### Background

Currently, there are over 30 drugs  on the market for treating epilepsy [64]. These ASMs are used as first line, adjunctive, and combination treatments, creating substantial heterogeneity in potential treatment regimens. In addition to these ASMs, alternative therapies including the ketogenic diet, vitamin supplementation, and surgical procedures can be employed to reduce seizure burden and improve QoL.

#### Knowledge gaps

Despite the numerous potential treatment options, there is little known about the optimal treatment regimens for individuals with SCN8A-RD [6, 28]. There are multiple potential sources of data that can inform clinical management, such as data collected in the Registry [4] and electronic medical records (EMRs). However, these sources collect seizure frequency data only a few times a year, making it difficult to generate high-quality, high-resolution longitudinal profiles documenting seizure frequency changes associated with changes in medication regimens. These limitations also reduce the potential of new and promising machine learning (ML) approaches that can handle large amounts of data to identify trends in medication response [65, 66]. Therefore, this WG focused on discussion of a unified infrastructure to allow for efficient collection of high-quality multisource data that would provide the framework for improved understanding of the efficacy of single ASMs, combinations of ASMs, alternative therapies, and transformative therapeutics.

#### Research priorities

The first priority is to design a strategy to collect granular data on seizure frequency in a prospective manner to advance knowledge about optimal medication combinations. The recommended procedure is to collect daily seizure data via a smartphone app to reduce the burden placed on caregivers. The minimum number of daily questions to be answered with a simple yes/no/number include: (1) did your child experience a seizure today, (2) if yes, how many, and (3) how many rescue medications were administered, if any?

Additional simple questions that could be incorporated into an electronic diary include the seizure type(s), sleep quality, and confirmation that the daily regimen of ASMs had been administered. Caregivers would also be prompted to provide more details on changes in development skills, QoL measures, etc. in an extended survey, which could be requested less frequently. The WG recommended the use of a well-vetted commercial application for seizure tracking with backend data processing capabilities [67]. The focus of the work would then be on efforts to merge the daily data with other sources of data, including those that come from analysis of biofluids (see WG4).

The second priority is to design a strategy to collect multisource data in a standardized format that would be deposited into an accessible repository. Since much of the data infrastructure already exists, albeit isolated in separate locations, it was proposed that a single repository host the data to more easily cross-reference and curate specific datasets on request.

There are additional advantages to the creation of this data infrastructure, including easy access of high-quality data for each patient prior to treatment, and enhanced attractiveness of the SCN8A-RD community for clinical trials. Such trials will have the capacity for more refined statistical designs, more appropriate inclusion criteria, and greater capacity to measure success. This infrastructure could potentially serve as a blueprint for other rare disease communities and enable better cross-disease studies.

### WG4: Application of biomarkers: tracking disease progression and treatment response

#### Background

The development of pharmaceutical agents and devices to treat, cure, and prevent epilepsy is greatly benefited by the identification of definitive biomarkers capable of reducing the cost of discovery and validation of new therapies for epilepsy. In principle, biomarkers may facilitate the development of interventions to prevent epilepsy, reverse progression of epilepsy, and potentially even cure epilepsy after it is established [68, 69]. Biomarkers may also play a role in identifying and effectively treating refractory epilepsy.

#### Knowledge gaps

While some biomarkers exist in the broader field of epilepsy, such as EEG-based markers and neurofilament light chain [68, 70–72], there are substantial gaps in biomarker availability for SCN8A-RD. Challenges include: (1) a lack of predictive biomarkers reflecting disease progression and treatment response, (2) limited understanding of the correlation between potential biomarkers and clinical outcomes, (3) technical challenges in developing highly sensitive and specific biomarker assays, and (4) regulatory hurdles in biomarker validation, particularly concerning FDA requirements.

#### Research priorities

To address these gaps, this WG discussed a multi-phased biomarker discovery and validation study. The initial discovery phase should focus on broad data collection from patients with pathogenic *SCN8A* variants, utilizing protein/cytokine/immune panels and omics-based approaches to analyze biofluids (e.g., blood and cerebrospinal fluid). Concurrently, advanced neuroimaging and EEG analysis should be conducted to identify potential biomarkers linked to seizure activity and disease progression [70]. ML models could be employed to integrate multisource data, aiming to identify complex biomarker signatures that may not be apparent through traditional analysis methods [73].

Rigorous validation would then be needed to determine which biomarkers faithfully reflect disease progression, treatment efficacy, and overall patient improvement. Special attention should be given to exploring Transcranial Magnetic Stimulation (TMS) has potential for measuring cortical excitation/inhibition ratios and ASM pharmacodynamics, which could provide valuable insights into disease mechanisms and treatment responses [74, 75].

Partnering with biotech and pharmaceutical companies could be initiated to accelerate biomarker discovery and integration into drug development pipelines. Patient and family engagement will be important for sample collection and to ensure that patient-reported outcomes are considered in biomarker validation. The creation of a centralized repository for biomarker data would facilitate data sharing and collaboration among researchers worldwide, potentially accelerating discoveries and validation processes.

### WG5: whole brain/whole body: brain regions, cell types, circuitry, neuromodulation an epileptic body

#### Background

The Na_V_1.6 channel is expressed in excitatory and inhibitory neurons throughout the CNS, as well as in many organs and cell types outside the brain [76]. *SCN8A* expression increases following birth and maintains expression in multiple brain regions commonly associated with epilepsy, such as the hippocampus, cortex, and thalamus [77]. These regions interact through circuitry networks that regulate cognition and behavior [78]. Little is understood about the impact of pathogenic *SCN8A* variants on whole-brain dynamics, including network synchronization and long-term neuroplasticity, and how these alterations govern the complex symptomatology observed in affected individuals. Moreover, there is uncertainty on whether many non-seizure outcomes are associated with central or peripheral aspects of SCN8A expression [79].

#### Knowledge gaps

This WG identified several knowledge gaps relating to genotype–phenotype relationships at the level of circuit biology and cellular physiology. Investigation is needed on the specific impact *SCN8A* variants have on different brain regions and on critical brain circuits, such as the thalamocortical network, and how these alterations contribute to seizure initiation and propagation. The differential impact of pathogenic SCN8A variants with GoF and LoF effects on various neuronal subtypes and how these contribute to overall network dysfunction are not fully elucidated, nor is the impact of such variants on physiology of organs and cells outside of the central nervous system. The role of glial cells, particularly astrocytes and microglia, in modulating the hyperexcitability caused by pathogenic *SCN8A* variants is poorly understood. Moreover, the mechanisms underlying the variability in treatment response are not well-characterized, hindering the development of personalized therapeutic approaches. Finally, there is a need to understand how other genes can modify SCN8A-RD phenotypes and whether targeting such modifiers may have therapeutic potential.

#### Research priorities

A primary goal is to perform detailed electrophysiological studies using both animal models and human tissue samples. In mouse models, single neuron measurements should be conducted to investigate how changes in Na_V_1.6 channel properties associated with both GoF and LoF variants lead to alterations in neuronal excitability in both excitatory and inhibitory neuronal systems. Patch-clamp recordings from brain slice preparations can provide crucial insights into how these variants affect neuronal firing patterns and synaptic transmission.

The second priority is to complement mouse studies by using CRISPR/Cas9 genome editing to create isogenic engineered *SCN8A* and control human iPSCs (hiPSCs). Expanded use of hiPSCs derived from patients offers the opportunity to study the effects of pathogenic variants on different human genetic backgrounds. Single-cell transcriptomics and multi-electrode array recordings of these neurons could reveal cell type-specific alterations in gene expression and network activity. Human iPSC models also provide an excellent platform for high-throughput screening of compound libraries to identify potential variant- or patient-specific therapeutic candidates.

The third goal is to investigate the role of different brain cell types, including neurons and glia, in both mouse and human models. This could involve using cell type-specific manipulations in mouse models and generating different neural cell types including the more complex brain organoids from human iPSCs. Single-cell RNA sequencing on these organoids would help to identify alterations in pathway activation. Further work using immune-altered mice could also allow for the study of human-derived neurons from hiPSCs transplanted and networked into the mouse brain.

The fourth goal is to map brain regions and circuits involved in epileptogenesis. Advanced in vivo electrophysiology techniques, such as multi-site recordings in freely behaving animals, can provide insights into how *SCN8A* variants impact circuit dynamics and seizure propagation. These studies should be complemented by ex vivo investigations using brain slice preparations, which allow for detailed examination of local circuit properties. The hiPSC derived brain organoids can also contribute to our understanding of basic circuits, where more advanced brain region specific organoids can elucidate novel network dynamics.

A fifth priority is to study whole brain dynamics using advanced neuroimaging approaches in both animal models and human patients. High-resolution structural and functional MRI, as well as diffusion tensor imaging, can map alterations in brain connectivity. These imaging approaches should be combined with transcranial magnetic stimulation, EEG and MEG studies to understand how SCN8A variants affect brain-wide synchronization and oscillatory patterns.

## Discussion

### Integration of stakeholder perspectives

The SCN8A Research Roadmap Meeting brought together global stakeholders to address critical knowledge gaps and to establish research priorities for improving clinical care of patients suffering with SCN8A-RD. Through comprehensive surveys of caregivers, clinicians, and scientists, and representatives of biotech, this initiative illuminated both challenges and opportunities in advancing therapeutic development and patient care. Coordinated surveys highlighted the desires of caregivers for improved seizure control and better management of non-seizure phenotypes. The promise of genetic therapies interested a good portion of the caregiver community, despite the length of time before such therapies would become available. Clinicians also tended to balance shorter-term improvements in care with longer-term research objectives. While clinicians were positive on the shorter-term potential of current therapeutics via improved application of polypharmacy, repurposing of drugs, and new evidence-based off-label treatments, scientists prioritized research aimed at understanding basic disease mechanisms with the primary goal of improving seizure control through the development of transformative therapeutics. Scientists and clinicians also highlighted the importance of interdisciplinary collaborations to advance understanding of disease mechanisms and management of seizure-related and non-seizure outcomes. Biotech representatives agreed with the potential of these varied research efforts, emphasizing the importance of natural history studies, biomarker discovery, variant classification, and measurements of non-seizure phenotypes.

The workshop also highlighted areas where the research community could be more responsive to caregiver needs. These include placing greater emphasis on non-seizure outcomes, QoL measures, and considering a broader range of treatment options. Differences between caregiver priorities and current research focus areas echo findings from other rare epilepsy communities [80–84]. It was acknowledged that current efforts to expand research to improve the understanding and management of non-seizure phenotypes is limited by current animal models and cellular systems.

### Interconnected priorities

The working groups identified several interconnected priorities that align with those identified in other epilepsy research initiatives, such as the EpiPM Consortium [85] and the Epilepsy Learning Healthcare System [86]. A key research priority of WG1 to address the "Goldilocks" challenge [87] parallels challenges faced in other channelopathies, especially among those with phenotypes associated with GoF and LoF variants [88]. The need to develop better understanding and treatment strategies for non-seizure outcomes discussed in WG2 [89] is shared in many other epilepsies that are associated with movement disorders, communication difficulties, and behavioral issues [84, 85, 89–91]. The proposed infrastructure for multisource data integration put forward by WG3 builds upon successful models from other rare epilepsies [92–94], which would be enhanced by the discovery of reliable biomarkers (WG4). Such efforts also align with WG1 priorities, as well as with the broader initiative to identify biomarkers in other epilepsy syndromes [68–72, 95]. The whole brain/whole body perspective advanced in WG5 represents a parallel effort that synergizes with the goals of other working groups striving to improve seizure and non-seizure outcomes. The comprehensive strategy proposed by WG5 to determine the functional roles played by *SCN8A* across different cell types, brain regions, and body systems has dual has potential to reveal cellular and neurophysiological aspects of brain function [41, 42, 95–100], as well as mechanisms underlying non-seizure phenotypes and effects on other organs. While most studies of mouse models have focused mainly on seizure burden and early death, some have demonstrated non-seizure phenotypes observed in patients, including impaired gait, tremor, ataxia, motor learning and coordination, deficits in associative learning or memory, hyperactivity, and impairments in social discrimination [31, 35, 39, 101, 102]. While seminal research demonstrated that global knockout of Scn8a in mice was associated with motor impairment and other deficits (1), a recent  conditional knockout of Na_v_1.6 specifically in peripheral sensory neurons was shown to result in severe ataxia and other motor coordination impairments [79]. Further research utilizing similar approaches promises to reveal the extent to which knockin variants with GoF or mixed GoF/LoF properties contribute to these impairments. This will also help to dissect the involvement of the central *versus* peripheral nervous system in non-seizure outcomes.

### Implementation challenges and opportunities

Several challenges must be addressed to successfully implement this roadmap. Some of the goals require sustained collaboration among members of different disciplines, for example, in the standardization and transmittal of medical data from clinicians to data scientists. New collaborative networks and research tools are needed to advance the study of non-seizure outcomes, including the recruitment of researchers with expertise in methods to develop and characterize animal models recapitulating these phenotypes. There is a need for increased research funding to understand SCN8A-RD and related epilepsies [103]. For severely affected individuals, the development of appropriate outcome measures will require new methodological approaches [81, 86, 104]. For some of the highlighted projects, funding mechanisms may already exist, while for others it will be necessary to seek novel funding sources. These challenges are balanced by significant opportunities that emerge from patient-centric collaborative research initiatives among laboratories and clinics, and that can be broadened to include cross-disease comparisons [105, 106]. Promising templates for transformative therapeutics are represented by advances in genetic therapies for SMA [107] and Dravet syndrome [108], and by other innovative modalities [109].

## Conclusion

This research roadmap workshop delineated a series of steps to accelerate research progress toward improving the health and well-being of patients with SCN8A-RD. This effort is especially well-timed given the urgent needs of patients and caregivers and the longer-term time frame required to translate research discovery into clinical practice. Importantly, the key stakeholders worked together to ensure incorporation the priorities of the caregiver community. Success in implementing these priorities over the next several years could significantly improve both seizure and non-seizure outcomes and provide a model for similar initiatives in other rare diseases. While the ultimate goal is to cure the disease, it was recognized that charting a realistic series of shorter-term benchmarks will help to monitor progress and set realistic expectations of the community of caregivers and patients. The comprehensive framework established by the roadmap effort serves to guide the research community to efficiently reach these critical goals.

## Supplementary Information

Additional file1 (PDF 2193 KB)

## Data Availability

The datasets supporting the conclusions of this article are included within the article and the supplementary figures. Additional information may be available from the corresponding author on reasonable request.
