# Supplementary material for: A research roadmap for SCN8A-related disorders: addressing knowledge gaps and aligning research priorities across stakeholders
Source: Orphanet J Rare Dis. 2025 Aug 19;20:444. doi: 10.1186/s13023-025-03672-w (PMC12366098; doi:10.1186/s13023-025-03672-w)
Supplement: Supplementary file 1 — Additional file1 (PDF 2193 KB) [file 13023_2025_3672_MOESM1_ESM.pdf]

Supplementary Figures

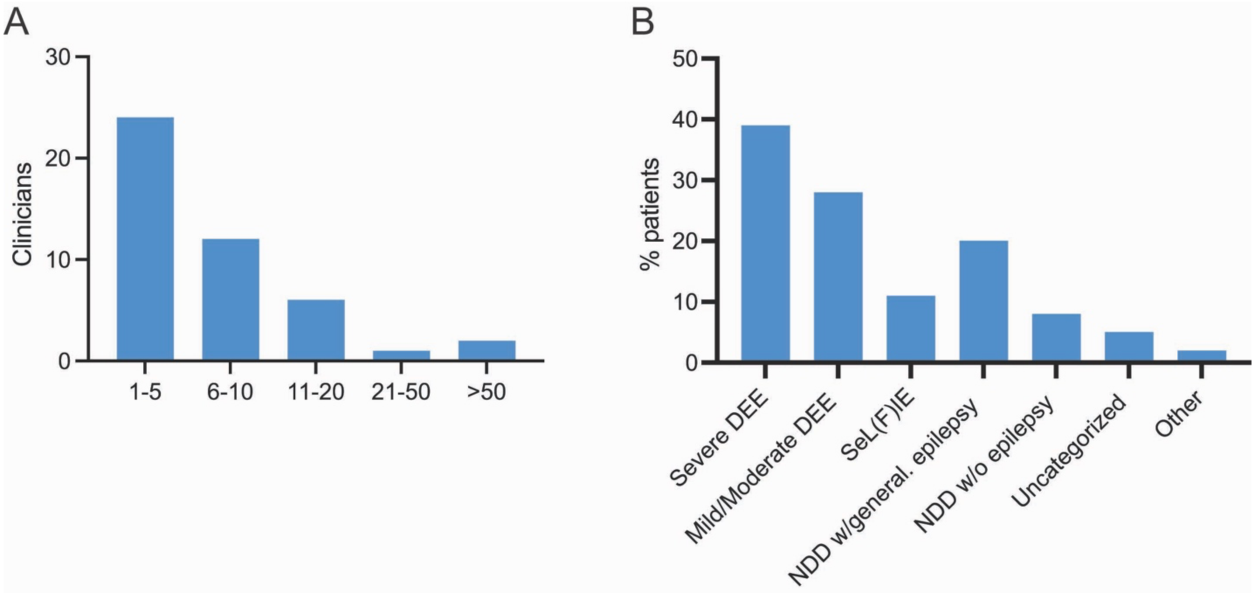

Fig S1. Clinician Experience in SCN8A-RD: Number of patients (A) and patient phenotypes (B).

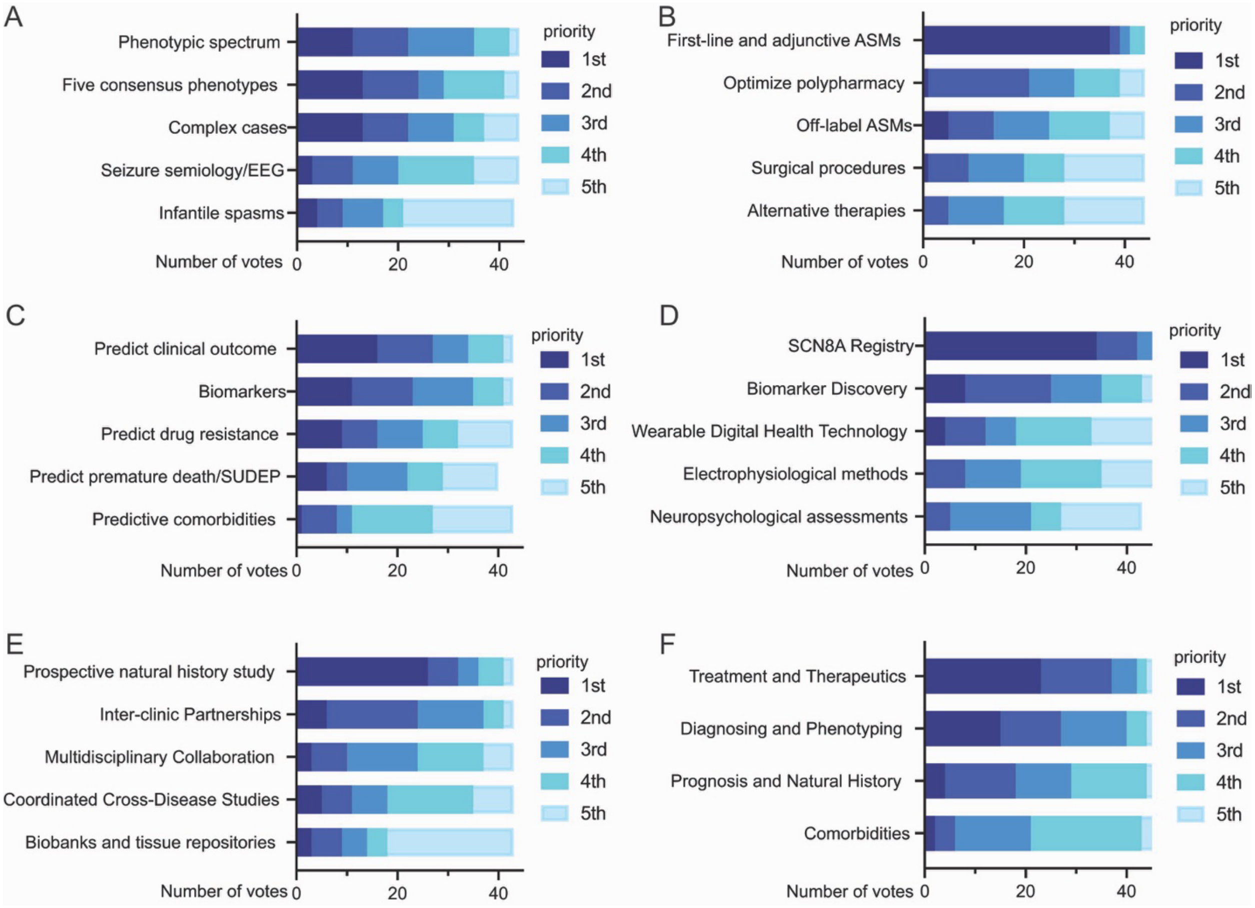

Fig S2. Clinician survey responses: A) Diagnosis and Phenotyping, B) Treatment, C) Prognosis, D) Comorbidities, E) Research tools, and F) Interdisciplinary research.

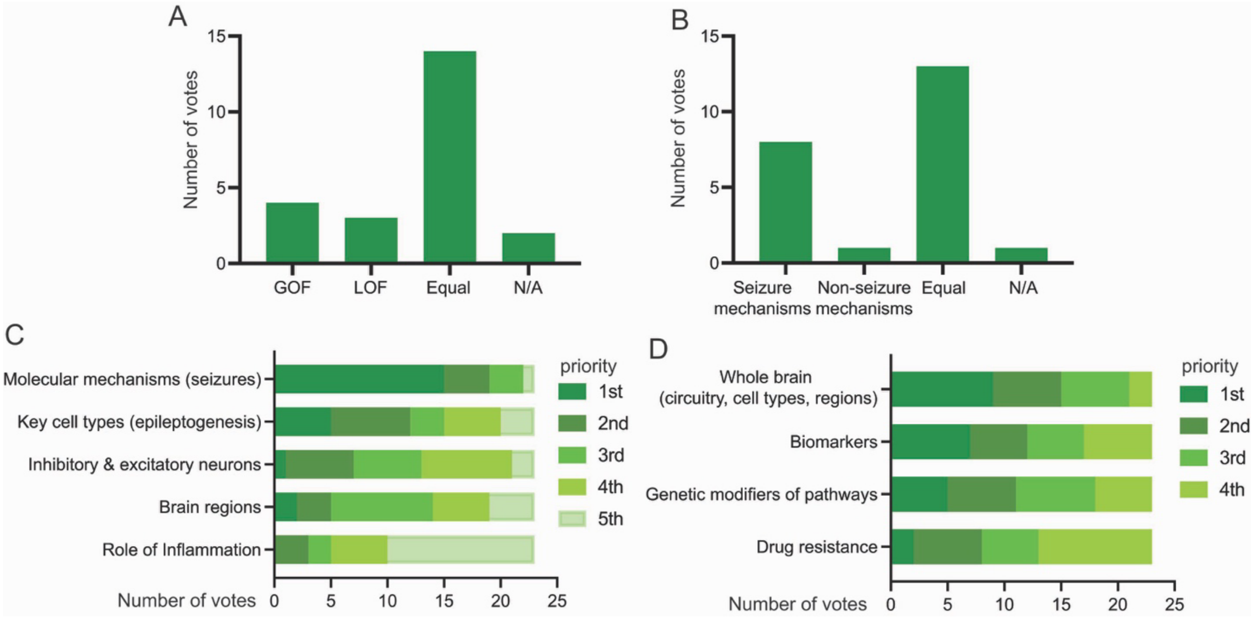

Fig S3. Disease mechanism research priorities of laboratory scientists: A) Feasibility of GOF *versus* LoF studies, B) seizure *versus* non-seizure related mechanisms, C) importance of cellular mechanisms, and D) importance of increased animal model research to advance therapeutic understanding of disease mechanisms.

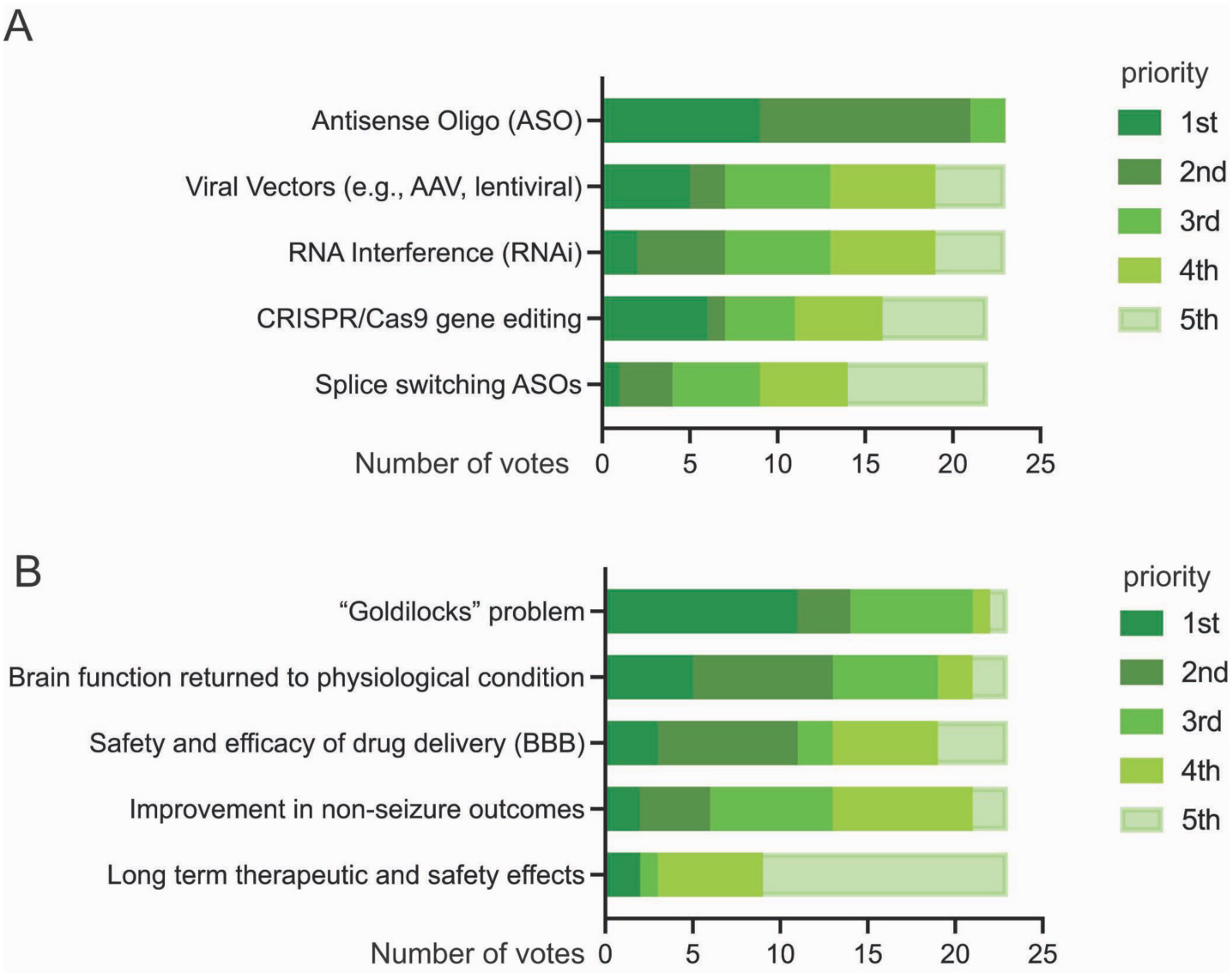

Fig S4. Gene Targeted Approaches: A) Priorities for GOF models and B) top challenges.

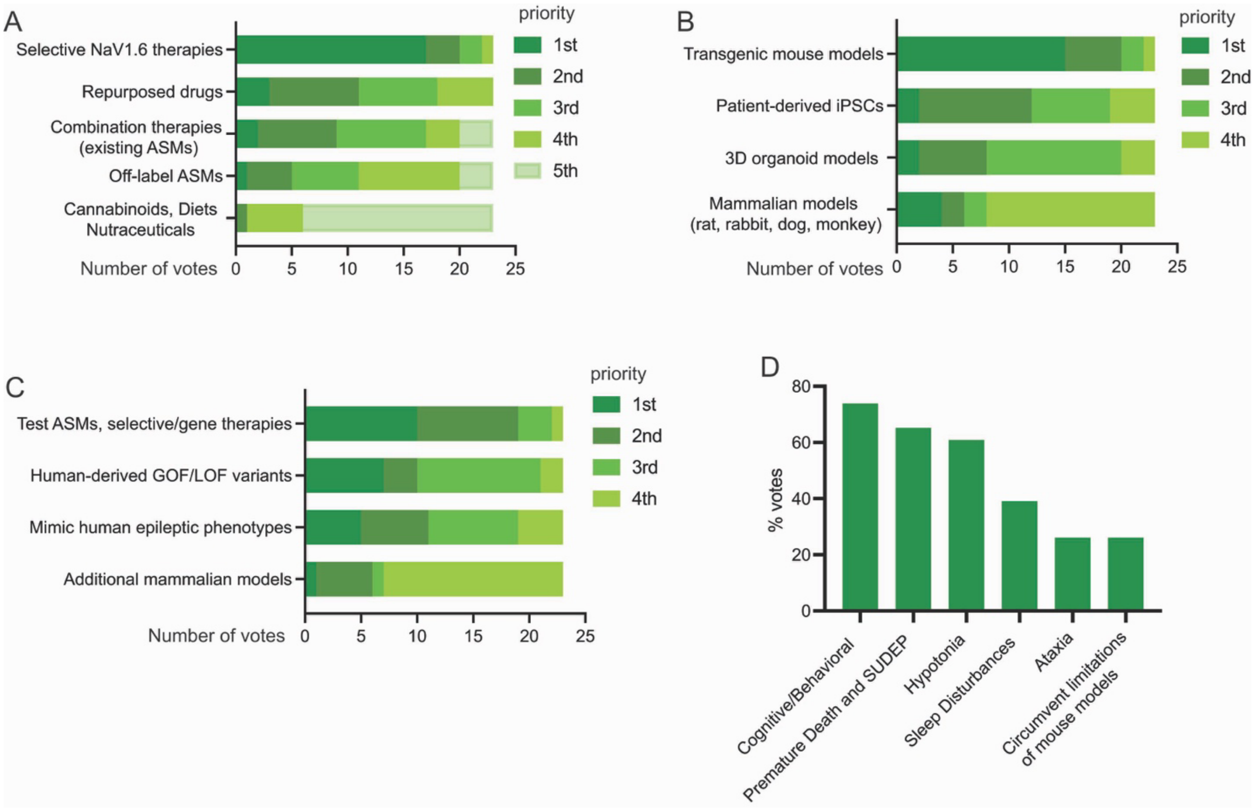

Fig S5. Laboratory models for disease mechanism and therapeutics studies: A) therapies types, B) existing model categories, C) new animal models for seizure, and D) non-seizure phenotypes.
